# Genetic pathogenesis, diagnosis, and treatment of short-chain 3-hydroxyacyl-coenzyme A dehydrogenase hyperinsulinism

**DOI:** 10.1186/s13023-021-02088-6

**Published:** 2021-11-04

**Authors:** Wei Zhang, Yan-Mei Sang

**Affiliations:** 1 Medizinische Klinik and Poliklinik IV, Klinikum der Universität München, LMU München, Munich, Germany; 2 Department of Pediatric Endocrinology, Genetic and Metabolism, Beijing Children’s Hospital, Capital Medical University, National Center for Children’s Health, Beijing, China

**Keywords:** Congenital hyperinsulinism (CHI), Hypoglycemia, Short-chain 3-hydroxyacyl-coenzyme A dehydrogenase hyperinsulinism (SCHAD-HI), Hydroxyacyl-coenzyme A dehydrogenase (HADH) gene

## Abstract

Congenital hyperinsulinism (CHI), a major cause of persistent and recurrent hypoglycemia in infancy and childhood. Numerous pathogenic genes have been associated with 14 known genetic subtypes of CHI. Adenosine triphosphate-sensitive potassium channel hyperinsulinism (KATP-HI) is the most common and most severe subtype, accounting for 40–50% of CHI cases. Short-chain 3-hydroxyacyl-coenzyme A dehydrogenase hyperinsulinism (SCHAD-HI) is a rare subtype that accounts for less than 1% of all CHI cases that are caused by homozygous mutations in the hydroxyacyl-coenzyme A dehydrogenase (HADH) gene. This review provided a systematic description of the genetic pathogenesis and current progress in the diagnosis and treatment of SCHAD-HI to improve our understanding of this disease.

Congenital hyperinsulinism (CHI) is a major cause of persistent and recurrent hypoglycemia in infancy and childhood with high genetic and clinical heterogeneity. At least 15 CHI-related pathogenic genes have been identified to date (ABCC8, KCNJ11, GLUD1, GCK, HADH, SLC16A1, UCP2, HNF1A, HNF4A, HK1, PGM1, PMM2, FOXA2, CACNA1D, and EIF2S3) that are associated with 14 known genetic subtypes of CHI [1]. Adenosine triphosphate (ATP)-sensitive potassium channel hyperinsulinism (KATP-HI) is the most common and most severe subtype [2], accounting for 40%-50% of CHI cases. Glutamate dehydrogenase hyperinsulinism (GDH-HI) accounts for ~ 5% of all CHI cases. All other subtypes of CHI are extremely rare [3]. Short-chain 3-hydroxyacyl-coenzyme A dehydrogenase hyperinsulinism (SCHAD-HI), caused by homozygous mutations in the hydroxyacyl-coenzyme A dehydrogenase (HADH) gene that lead to deficient SCHAD activity, is a rare CHI subtype accounting for less than 1% of all cases. However, SCHAD-HI is a common CHI subtype observed in consanguineous marriages [4]. To date, approximately five pedigrees including at least 45 patients with SCHAD-HI have been reported. This review provides a systematic description of the genetic pathogenesis and current progress in the diagnosis and treatment of SCHAD-HI to improve clinicians’ understanding of this disease.

## SCHAD-HI

Hyperinsulinism caused by recessive mutations in the HADH gene was first reported by Clayton et al. in 2001 [5]. The proband was a female infant born to a non-consanguineous Indian couple at 38 weeks gestation with a birth weight of 3.2 kg (kg). The infant experienced feeding difficulty immediately after birth. Hypoglycemia was diagnosed at a local hospital following convulsions at four months of age, and hyperinsulinemia was subsequently diagnosed at 14 months of age. Blood samples collected at the onset of hypoglycemia showed significantly elevated blood acetylcarnitine concentrations. In addition, an examination of organic acid metabolites in the urine showed slight increases in 3-hydroxybutyrate, 3-hydroxyglutarate, and 3,4-dihydroxybutyrate. Further examination of fatty acid metabolism showed that the activity of the short chain HADH enzyme in fibroblasts was significantly lower than those in normal controls. At 19 months of age, diazoxide (brand names: Proglycem®, Hyperstat) and hydrochlorothiazide (brand names: Aquazide H, Hydrocot, Microzide, Zide) were orally administered and demonstrated clinical effectiveness. Deoxyribonucleic acid (DNA) sequencing of family members revealed that the affected child carried a homozygous c.773C > T mutation in exon 7 of the HADH gene, which caused a proline (Pro) to leucine (Leu) substitution at the 258th amino acid (Pro258Leu) in one of the alpha-helices of the C-terminal domain of the SCHAD protein. Both parents were heterozygous for the Pro258Leu mutation.

## HADH gene and SCHAD protein

**Fig. 1:**
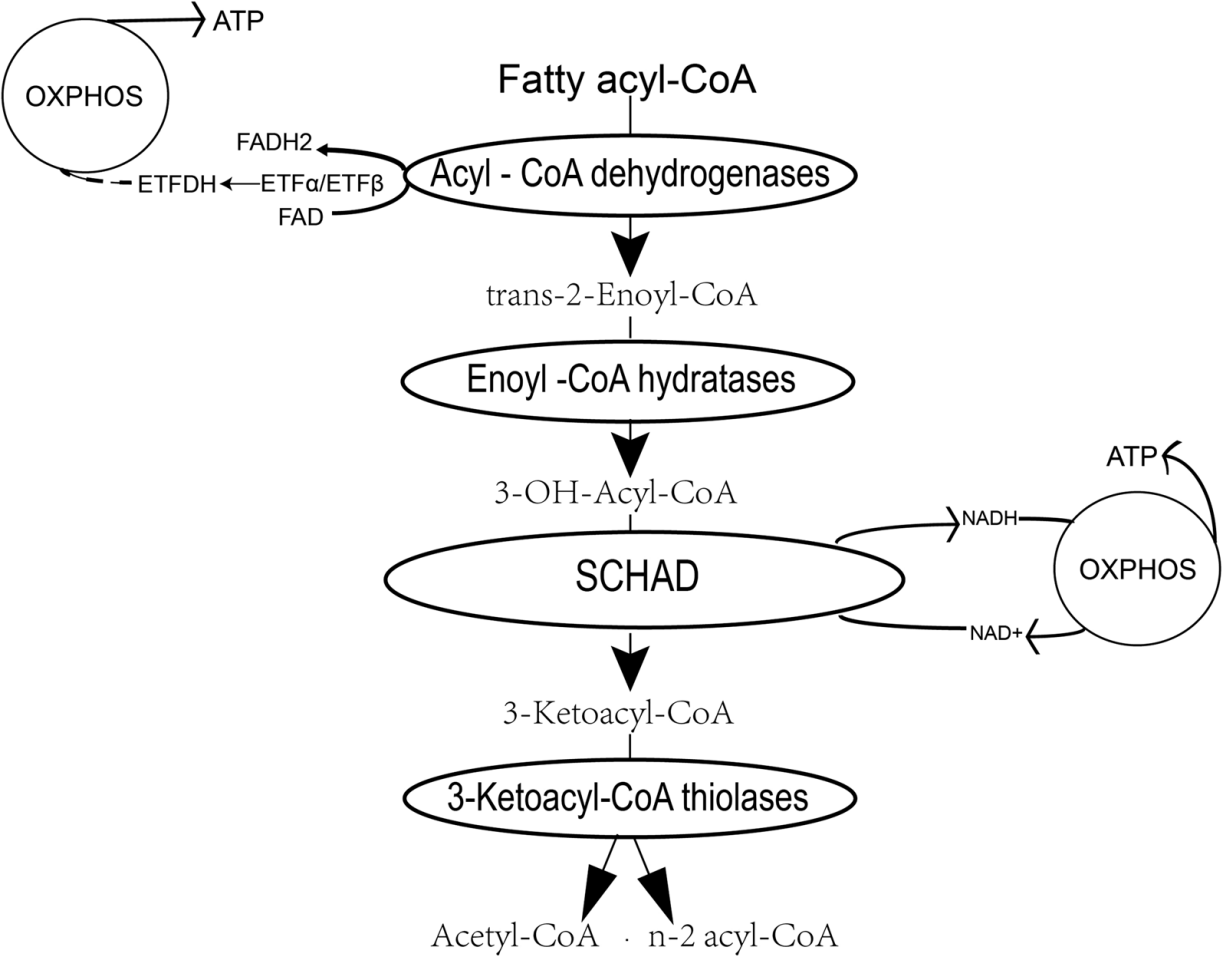
SCHAD is involved in fatty acidsβ-oxidation: Acyl-CoAs are degraded in mitochondria by four enzymatic steps of β-oxidation. The process of each cycle: the first step is dehydrogenation by FAD, the acyl-CoA dehydrogenase catalyzed acyl-CoA into trans-Δ^2^-enoyl-CoA. Then a hydration catalyzed by an enoyl-CoA hydratase happened, the product is 3-hydroxyacyl-CoA. During the third step, 3-hydroxyacyl-CoA is dehydrogenated into 3-ketoacyl-CoA by 3-hydroxyacyl-CoA dehydrogenase. In the end, thiolase cleaves 3-keto-CoA into a two-carbon chain shortened acyl-CoA and an acetyl-CoA. The process continues until all the carbon in the fatty acid has been converted to acetyl-CoA. *SCHAD* short-chain L-3-hydroxyacyl-CoA dehydrogenase; *ETFDH* electron transfer flavoprotein-ubiquinone oxidoreductase; *OXPHOS* oxidative phosphorylation system

The HADH gene is located on chromosome 4q25, spanning 49 kilobases (kb) and containing eight exons and seven introns (https://www.ncbi.nlm.nih.gov/gene/3033). HADH is a member of the 3-hydroxyacyl-CoA dehydrogenase gene family, which encodes SCHAD, also known as type 10 17β-hydroxysteroid dehydrogenase (HSD10) [6], a mitochondrial enzyme composed of 314 amino acids. Mitochondria are the major sites of energy metabolism in eukaryotic cells. Fatty acid oxidation (FAO) takes place through four steps of enzymatic reactions, including dehydrogenation, hydration, dehydrogenation again, and thiolytic cleavage [7]. Through these catalytic actions, fatty acids are oxidized and degraded to CO_2_ and H_2_O, while releasing energy in the form of ATP. SCHAD enzyme is a component of the enzymatic reaction described above [8]. It catalyzes the penultimate reaction in β-oxidation of fatty acids (L3-hydroxyacyl-CoA + NAD^+^  → β-ketoacyl-CoA + NADH + H^+^) in the mitochondrial matrix [9], where it dehydroxylates medium- and short-chain NAD^+^-dependent L3-hydroxyacyl-CoAs to 3-ketoacyl-CoA and NADH, respectively (Fig. 1) [10]. Besides, SCHAD is expressed at higher levels than other fatty acid β oxidases including acyl-CoA dehydrogenase and acetyl-CoA acyltransferase 2 [11]. The enzymatic activity of SCHAD is the most efficient for metabolizing medium-chain-length fatty acids [12].

SCHAD is expressed in multiple tissues throughout the human body including adipocytes, kidneys, and lymphoblasts. It shows the highest level of expression in pancreatic beta cells (http://biogps.org/#goto=genereport&id=3033). The two main physiological roles of SCHAD within the human body include fatty acid metabolism and mediating insulin secretion. When hepatic glycogen is depleted from the human body due to malnutrition (limited carbohydrate intake) or fasting, the fatty acid β-oxidation pathway will become the primary energy source. More than 80% of the energy supply for the heart and liver is derived from fatty acid β-oxidation [13]. Therefore, decreased SCHAD expression, due to mutations in the HADH gene, will lead to a reduction in medium- and short-chain fatty acid metabolism, resulting in a state of energy (ATP) deficiency with negative impacts on the human body.

## Mutation profiles of SCHAD-HI related genes

At least five pedigrees have been reported to date with more than 45 cases of autosomal recessive SCADH-HI. Approximately 25 mutations have been reported, according to the Human Gene Mutation Database (HGMD, http://www.hgmd.org/; Cardiff, UK), including 11 missense or nonsense mutations, five deletions, seven splice site mutations, one insertion, and one chromosomal cross-over deletion mutation (Table 1) [4, 14–23]. The typical c.636 + 471G > T splicing mutation is an intron splice site mutation that introduces a cryptic splice donor site that leads to pseudo activation and generates a premature termination codon. In addition, our group previously reported one case with compound heterozygous mutations at c.419 + 1G > A and c.547-1G > C in an intronic region of the HADH gene [24]. The c.419 + 1G > A mutation, which has the potential to be pathogenic because it is located in a splice site of the HADH gene, was already present in the SNP database. The c.547-1G > C mutation had not been previously reported and has not yet been included in the SNP database. Functional studies for these two splice site mutations predict a deleterious effect on HADH splicing and provide strong evidence for pathogenicity.

**Table 1:** Reported mutated types of HAHD gene in SCHAD-HI patients

Mutation type	Acid change (nuleotide change)	
Missense/nonsense	p.R10P (c.29G > C)	p.I184F(c.550A > T)
	p.V30E (c.89T > A)	p.Y226H(c.676 T > C)
	p.G34A (c.100G > C)	p.R236X(c.706C > T)
	p.D57G(c.170A > G)	p.P258L (c.773C > T)
	p.K136E (406A > G)	p.M188V (c.562A > G)
	p.Q163X (c.487C > T)	
Splice	p.(c.133-1G > A)	p.(c.710-2A > G)
	p.(c.419 + 1G > A)	p.(c.709 + 39C > G)
	p.(c.547-1G > C)	p.(c.261 + 1G > A)
	p.(c.636 + 471G > T)	
Small deletions	p.Val116Trpfs*9(c.346delG)	p.S196fs (c.587del)
	p.T189fs (c.565del)	p.K206fsX14 (c.617delA)
	p.(c.547-3_549del)	
Deletions/indels	p.K95S (c.283_293del11insT)	
Cross deletions	p.(c.1-3440_132 + 1943 [ex. 1])	

## Genetic pathogenesis and histological types of SCHAD-HI

In pancreatic tissues, the highest levels of SCHAD enzymatic activity have been detected in pancreatic islets, especially in pancreatic beta cells, indicating that fatty acid oxidative metabolism may play an important role in the process of insulin release [25]. However, the specific molecular mechanisms by which HADH mutations lead to abnormal insulin release remain unknown. Filling et al. reported a direct protein–protein interaction between SCHAD and glutamate dehydrogenase (GDH), through which SCHAD may affect GDH activity and insulin release [26].

**Fig. 2:**
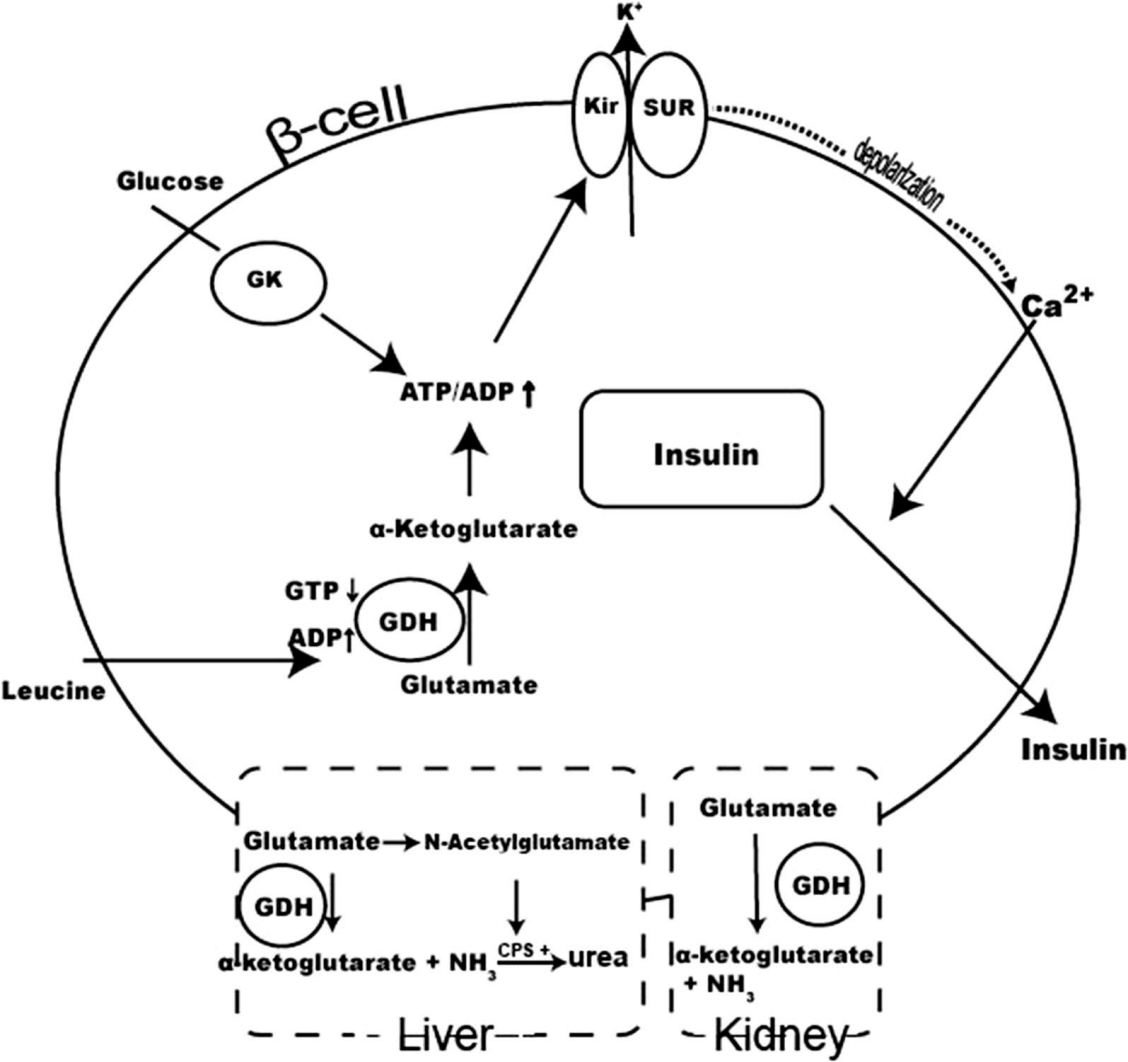
Mechanisms of GHD-HI [30]. In β-cells, oxidation of glucose increases the ATP/ADP ratio, which inhibits ATP-dependent potassium channels, triggering the opening of voltage-gated calcium channels and the inward flow of calcium to allow insulin to be released. Amino acids enter this triggered pathway through the oxidation of glutamate by glutamate dehydrogenase (GDH) under the regulation of GTP and ADP and leucine. In the liver, ammonia can be produced from glutamate via GDH; glutamate produces N-acetylglutamate to manage the detoxification of ammonia to urea as well. In the kidney, GDH catalyses the conversion of glutamate into alpha-ketoglutarate and ammonia; this process is thought to be responsible for the hyperammonemia in HI/HA syndrome. *GDH* glutamate dehydrogenase, *GK* glucokinase, *SUR* sulfonylurea receptor; *KATP channel* ATP-dependent potassium channel; *Kir* potassium inward rectifying pore; *CPS* carbamoyl-phosphate synthetase

GDH is a mitochondrial enzyme encoded by the GLUD1 gene that catalyzes the oxidative deamination of glutamate to α-ketoglutarate and ammonia [27]. α-ketoglutarate is an intermediate metabolite of the tricarboxylic acid (TCA) cycle that stimulates insulin release in pancreatic beta cells (Fig. 2). GDH can be allosterically activated by leucine and adenosine diphosphate (ADP), but be inhibited by guanosine-5'-triphosphate (GTP) [28]. GLUD1 missense mutations reduce the sensitivity of GDH to allosteric inhibition by GTP and ATP, which results in an increase in glutamate deamination induced by leucine. As a result, increased glutamate deamination may produce a protein sensitive hyperinsulinism-hyperammonemia syndrome (HI/HA) in which patients experience hypoglycemia after excessive leucine intake [29].

**Fig. 3:**
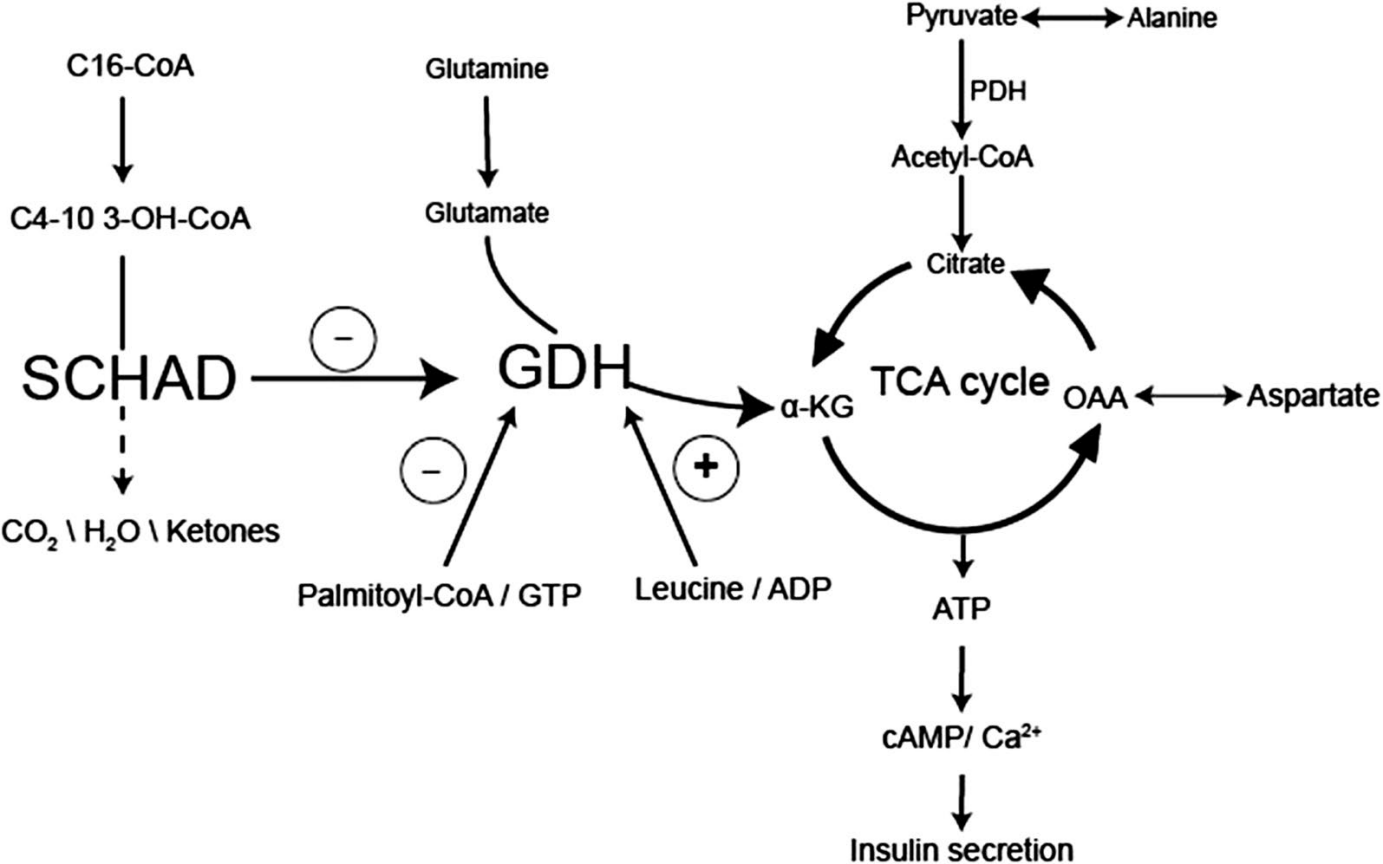
Mechanisms of insulin dysregulation in SCHAD deficiency. In SCHAD-deficient islets, loss of the inhibitory protein–protein interaction of SCHAD on GDH results in increased sensitivity of GDH to allosteric activation of leucine. Glutamine plus leucine has the strongest stimulatory effect on islets in vitro assays. On top of glutamine plus leucine stimulation, increasing alanine secondary to more insulin release, as the TCA cycle can produce more ATP. *GDH* glutamate dehydrogenase, *SCHAD* short-chain 3-hydroxyacyl-CoA dehydrogenase, *α-KG* α-ketoglutarate, *OAA* oxaloacetate, and *TCA* tricarboxylic acid

Under normal physiological conditions, SCHAD may perform different functions in multiple metabolic pathways. SCHAD can exert its moonlighting protein effects through which SCHAD may directly inhibit GDH enzymatic activity in pancreatic beta cells by affecting the carboxyl binding of substrates at the catalytic site, which decreases the affinity of GDH for α-ketoglutarate, a glutamate metabolite [31]. In 2010, Li et al. [32] reported that basal glucose levels significantly decreased and basal insulin increased in HADH knockout (KO) mice compared with wild-type after fasting 2 h as well as 24 h. Following oral amino acid, a significant decrease of glucose and elevation in insulin were also shown. In addition, GDH enzyme kinetics showed that affinity for GDH of α-ketoglutarate decreased in HADH KO mice. Importantly, they also found GDH co-precipitated with SCHAD in liver mitochondria of wild-type mice, but not in KO mice. This showed the protein–protein interactions between SCHAD and GDH. Loss of SCHAD moonlighting capabilities in SCHAD-deficient mice, generated by knockout of the HADH gene, increased GDH enzymatic activity, enhanced GDH sensitivity to its activator leucine, and impaired allosteric inhibition of GDH by GTP, resulting in increased oxidation of glutamate and elevated intracellular ATP production. An increase in the ATP/ADP ratio may lead to the closure of ATP-sensitive potassium (Adenosine triphosphate-sensitive potassium, KATP) channels on the cell membrane of pancreatic beta cells. The subsequent cessation of K^+^ efflux may result in depolarization of the cell membrane and the opening of voltage-gated Ca^2+^ channels. The subsequent influx of Ca^2+^ ions into pancreatic beta cells activates insulin secretion [33] (Fig. 3). Of note, patients with SCHAD-HI usually do not develop hyperammonemia [34], indicating that HADH mutations do not exclusively cause leucine sensitivity and dysregulation of insulin secretion through the GTP-regulated GDH pathway. Although specific mechanisms of function are not fully understood, some researchers have suggested that this phenomenon may be related to the high pancreatic expression of HADH and relatively low expression in other tissues such as the liver and kidney, causing the activation of GDH to be restricted to pancreatic islets [35].

Additional research has revealed that SCHAD deficiency also plays an important role in hyperinsulinemia induced by knockout of the islet specific transcription factor Fork head box A2 (FOXA2) gene. Intron 1 of the HADH gene is highly conserved and contains the binding site for Foxa2. Murine experiments have shown that Foxa2 can directly bind to HADH and activate its transcriptional activity [36]. Sund et al. [37] showed that Foxa2 knockout mice had a threefold downregulation of HADH mRNA levels compared with wild-type mice, which caused severe hyperinsulinemic hypoglycemia. Similarly, Mary et al. [38] showed a 25.7% reduction in levels of SCHAD protein expression in a Foxa2 knockdown human embryonic kidney cell line (293 T cell line) compared with a wild-type cell line.

Patients with SCHAD-HI do not commonly undergo pancreatectomy, therefore, pancreatic pathology specimens from affected patients are scarce. All pancreatic samples reported to date from patients with SCHAD-HI are diffuse [39].

## Clinical characteristics of SCHAD-HI

All cases of SCHAD-HI reported to date are autosomal recessive, and most cases are observed in children with normal birth weights from consanguineous relationships. Patients most commonly present in early infancy, usually before 14 months of age. Few cases are characterized by onset in childhood or adulthood. The first known adult SCHAD-HI patient with a HADH mutation was 21 years of age but experienced disease onset at age one. In this patient, hypoglycemia gradually diminished with age [40].

Patients with SCHAD-HI exhibit clinical characteristics that are similar to GDH-HI and present with fasting-, leucine-, and protein-sensitive hypoglycemia. Children with SCHAD-HI show variability in clinical presentation and may present with late-onset mild hypoglycemia or severe neonatal hypoglycemia, usually without hyperammonemia. A large proportion of affected children exhibit abnormal fatty acid metabolism, with elevated concentrations of urinary 3-hydroxyglutarate and plasma 3-hydroxybutyrylcarnitine. However, Kapoor et al. [41] reported the case of a SCHAD-HI patient who carried a HADH c.562A > G (Met188Val) mutation but had normal plasma carnitine concentrations and negative urine organic acids in 2009. Long term follow-up studies indicate that children with SCHAD-HI usually have a normal liver function, without cardiomyopathy or other skeletal muscle metabolic disorders.

## Diagnosis of SCHAD-HI

Clinical diagnosis of SCHAD-HI is conducted using procedures similar to those used for other types of CHI. Criteria include (1) presentation with nonketotic hypoglycemia requiring a high fluid glucose infusion to control hypoglycemic episodes and (2) inappropriate insulin hypersecretion. Specific diagnostic indicators of CHI are available in the CHI guidelines (Table 2) [42]. After a CHI diagnosis has been confirmed, genetic testing may indicate SCHAD-HI based on the presence of a HADH gene mutation.

**Table 2:** Dignostic criteria for congenital hyperinsulinism

	Detected when plasma glucose < 2.8 mmol/L
Plasma insulin, plasma C-peptide	> 1–2uU/mL (insulin), > 0.2 mmol/L (C-peptide)
Plasma free fatty acids	< 1.7 mmol/L
Plasma β-hydroxybutyrate	< 1.8 mmol/L
Glycemic response to glucagon	≥ 1.7 mmol/L

## Treatment of SCHAD-HI

Treatment with high rate intravenous glucose infusion in the acute phase of SCHAD-HI and other types of CHI is recommended to prevent or slow the onset of hypoglycemia and avoid permanent neurologic damage in affected children [43]. Diazoxide is a KATP channel activator that has become the first-line pharmacologic agent for controlling hypoglycemic episodes in patients with CHI [44]. Because HADH mutations do not cause structural alterations to KATP channels, all known SCHAD-HI patients respond to diazoxide [45]. The effective dose of diazoxide in patients with SCHAD-HI ranges from 5–10 mg/kg/d [39]. The most clinically-important side effects of diazoxide treatment are water and sodium retention, which may require furosemide (brand name: Lasix) or other potent diuretics to prevent heart failure in infants who receive high-fluid glucose infusions to control hypoglycemia. Other side effects include neutropenia, hypervolemic hypertension, and hirsutism [46]. Among these side effects, fluid overload, edema and hirsutism are universal and dose dependent while the other side effects are idiosyncratic.

Octreotide (brand name: Sandostatin®) is currently used for off-label treatment if the patient is unable to continue taking diazoxide due to adverse side effects or other reasons [47]. Octreotide is a somatostatin analogue that has become the most popular second-line agent for treating CHI. Octreotide can inhibit insulin and glucagon release from pancreatic islet cells by binding to the somatostatin receptor 2 (SSTR2) and somatostatin receptor 5 (SSTR5) [48]. A case of SCHAD-HI in Saudi Arabia was effectively treated with octreotide because diazoxide was unavailable. This patient followed a low-protein diet for an extended period time and avoided prolonged fasting. Hypoglycemic episodes were not observed after discontinuation of octreotide as the patient aged [40]. Common side effects associated with octreotide use include gastrointestinal symptoms such as leukoaraioid stools, abdominal discomfort, cholecystitis, and gallstones. Less common but more serious complications include hypotension, abnormal liver functions, thrombocytopenia, hyperkalemia, long QT-gap syndrome, and leukocytosis. Prolonged administration of octreotide may result in growth retardation and potentially lethal necrotizing enterocolitis [49]. Licensed glucagon is only recommended off-label during intensive therapy, but long-term use of stable aqueous is currently in phase 2 clinical trial and nifedipine is not anymore recommended, due to inefficient glycemic effect [50].

For patients whose main manifestation of the disease is postprandial hypoglycemia, dietary regulation is critical. SCHAD-HI patients may develop hypoglycemia induced by consumption of a high-protein diet rich in leucine. Therefore, dietary modifications should include long-term carbohydrate, cellulose, and fat milk supplementation, avoiding high-protein diets and prolonged fasting. At present, no specific clinical recommendations govern the intake of relevant proteins.

## Prognosis of SCHAD-HI

Most patients with SCHAD-HI respond well to pharmacologic therapy with diazoxide. Therefore, if diagnosed and treated promptly, most patients can effectively avoid complications such as neurological damage due to persistent recurrent hypoglycemia and experience a positive clinical outcome. Due to the low incidence of SCHAD-HI and few clinically reported cases, few relevant studies have examined the self-remission rate.

In conclusion, SCHAD-HI is an extremely rare condition. Children with SCHAD-HI usually exhibit early disease onset, but respond well to diazoxide therapy. Children diagnosed with CHI are strongly recommended to undergo a cytogenetic examination and DNA-based genetic testing to optimize the treatment regimen and improve prognosis. Clinicians should be involved in creating a transitional clinical environment so that CHI patients may receive appropriate care from childhood to adulthood.

## Data Availability

Orphanet journal of rare disease remains neutral with regard to jurisdicional claims in published maps and institutional affiliations.

## Abbreviations

CHI: Congenital hyperinsulinism
SCHAD-HI: Short-chain 3-hydroxyacyl-coenzyme A dehydrogenase hyperinsulinism
HSD10: Type 10 17β-hydroxysteroid dehydrogenase
HADH: Hydroxyacyl-coenzyme A dehydrogenase
KATP-HI: Adenosine triphosphate-sensitive potassium channel hyperinsulinism
GDH-HI: Glutamate dehydrogenase hyperinsulinism
GDH: Glutamate dehydrogenase
TCA: Tricarboxylic acid
ADP: Adenosine diphosphate
GTP: Guanosine-5′-triphosphate
DNA: Deoxyribonucleic acid
HI/HA: Hyperinsulinism-hyperammonemia syndrome
FOXA2: Forkhead box A2
SSTR: Somatostatin receptor
GK: Glucokinase
SUR: Sulfonylurea receptor
Kir: Potassium inward rectifying pore
CPS: Carbamoyl-phosphate synthetase
Pro: Proline
Leu: Leucine
α-KG: α-Ketoglutarate
OAA: Oxaloacetate
Kb: Kilobase
Kg: Kilogram
ETFDH: Electron transfer flavoprotein-ubiquinone oxidoreductase
OXPHOS: Oxidative phosphorylation system

## References

[1] Gϋemes M, Rahman SA, Kapoor RR (2020). Hyperinsulinemic hypoglycemia in children and adolescents: recent advances in understanding of pathophysiology and management. Rev Endocr Metab Disord.

[2] Stanley CA (2002). Advances in diagnosis and treatment of hyperinsulinism in infants and children. J Clin Endocrinol Metab.

[3] Marquard J, Palladino AA, Stanley CA, Mayatepek E, Meissner T. Rare forms of congenital hyperinsulinism. Seminars in pediatric surgery; 2011: Elsevier; 2011. p. 38–44. 10.1053/j.sempedsurg.2010.10.006. 10.1053/j.sempedsurg.2010.10.006 21186003

[4] Flanagan SE, Patch AM, Locke JM (2011). Genome-wide homozygosity analysis reveals HADH mutations as a common cause of diazoxide-responsive hyperinsulinemic-hypoglycemia in consanguineous pedigrees. J Clin Endocrinol Metab.

[5] Clayton PT, Eaton S, Aynsley-Green A (2001). Hyperinsulinism in short-chain L-3-hydroxyacyl-CoA dehydrogenase deficiency reveals the importance of β-oxidation in insulin secretion. J Clin Investig.

[6] Yang SY, He XY, Schulz H (2005). 3-hydroxyacyl-CoA dehydrogenase and short chain 3-hydroxyacyl-CoA dehydrogenase in human health and disease. FEBS J.

[7] Wanders R, Vreken P, Den Boer M, Wijburg F, Van Gennip A, IJist L. Disorders of mitochondrial fatty acyl‐CoA β‐oxidation. J Inherit Metab Dis 1999; 22(4): 442–87. 10.1023/a:1005504223140. 10.1023/a:1005504223140 10407780

[8] Houten SM, Wanders RJ (2010). A general introduction to the biochemistry of mitochondrial fatty acid β-oxidation. J Inherit Metab Dis.

[9] Vredendaal PJ, van den Berg IE, Stroobants AK, van der AD, Malingre HE, Berger R. Structural organization of the human short-chain L-3-hydroxyacyl-CoA dehydrogenase gene. Mamm Genome 1998;9(9):763–8. 10.1007/s003359900860. 10.1007/s003359900860 9716664

[10] Houten SM, Violante S, Ventura FV, Wanders RJ (2016). The biochemistry and physiology of mitochondrial fatty acid β-oxidation and its genetic disorders. Annu Rev Physiol.

[11] Stanley CA, Bennett MJ, Mayatepek E (2006). Disorders of mitochondrial fatty acid oxidation and related metabolic pathways.

[12] Pepin E, Guay C, Delghingaro-Augusto V, Joly E, Madiraju SR, Prentki M (2010). Short-chain 3-hydroxyacyl-CoA dehydrogenase is a negative regulator of insulin secretion in response to fuel and non-fuel stimuli in INS832/13 β-cells. J Diabetes.

[13] Lopaschuk GD, Ussher JR, Folmes CD, Jaswal JS, Stanley WC (2010). Myocardial fatty acid metabolism in health and disease. Physiol Rev.

[14] Martins E, Cardoso ML, Rodrigues E (2011). Short-chain 3-hydroxyacyl-CoA dehydrogenase deficiency: the clinical relevance of an early diagnosis and report of four new cases. J Inherit Metab Dis.

[15] Kapoor RR, Flanagan SE, Arya VB, Shield JP, Ellard S, Hussain K (2013). Clinical and molecular characterisation of 300 patients with congenital hyperinsulinism. Eur J Endocrinol.

[16] Snider KE, Becker S, Boyajian L (2013). Genotype and phenotype correlations in 417 children with congenital hyperinsulinism. J Clin Endocrinol Metab.

[17] Flanagan SE, Xie W, Caswell R (2013). Next-generation sequencing reveals deep intronic cryptic ABCC8 and HADH splicing founder mutations causing hyperinsulinism by pseudoexon activation. Am J Hum Genet.

[18] Popa FI, Perlini S, Teofoli F (2012). 3-hydroxyacyl-coenzyme a dehydrogenase deficiency: identification of a new mutation causing hyperinsulinemic hypoketotic hypoglycemia, altered organic acids and acylcarnitines concentrations. JIMD Rep.

[19] Xu ZD, Zhang W, Liu M (2018). Analysis on the pathogenic genes of 60 Chinese children with congenital hyperinsulinemia. Endocr Connect.

[20] Kaur S, Kulkarni KP, Kochar IP (2010). Severe dietary protein sensitivity and hyperinsulinemic hypoglycemia in a patient with heterozygous mutation in HADH gene. J Pediatr Endocrinol Metab JPEM.

[21] Senniappan S, Sadeghizadeh A, Flanagan SE (2015). Genotype and phenotype correlations in Iranian patients with hyperinsulinaemic hypoglycaemia. BMC Res Notes.

[22] Satapathy AK, Jain V, Ellard S, Flanagan SE. Hyperinsulinemic hypoglycemia of infancy due to novel HADH mutation in two siblings. Indian Pediatr 2016; 53(10): 912–3. 10.1007/s13312-016-0958-1. 10.1007/s13312-016-0958-1 27771675

[23] Özsu E, Mutlu GY, Çizmecioglu FM, Hatun SJJoCRiPE. HADH mutation is a rare cause of hyperinsulinaemic hypoglycaemia. 2015; 7(1).

[24] Wei Z, et al. Clinical analysis of 1 case of congenital hyperinsulinemia caused by HADH gene mutation and literature review. Chin J Diabetes Mellitus, 2019. 011(002): p. 132–134. 10.3760/cma.j.issn.1674-5809.2019.02.009.

[25] Malaisse WJ, Malaisse-Lagae F, Sener A, Hellerstrom C (1985). Participation of endogenous fatty acids in the secretory activity of the pancreatic B-cell. Biochem J.

[26] Filling C, Keller B, Hirschberg D (2008). Role of short-chain hydroxyacyl CoA dehydrogenases in SCHAD deficiency. Biochem Biophys Res Commun.

[27] Plaitakis A, Kalef-Ezra E, Kotzamani D, Zaganas I, Spanaki C (2017). The glutamate dehydrogenase pathway and its roles in cell and tissue biology in health and disease. Biology (Basel).

[28] Fahien LA, Macdonald MJ (2011). The complex mechanism of glutamate dehydrogenase in insulin secretion. Diabetes.

[29] Kelly A, Ng D, Ferry RJ (2001). Acute insulin responses to leucine in children with the hyperinsulinism/hyperammonemia syndrome. J Clin Endocrinol Metab.

[30] Palladino AA, Stanley CA (2010). The hyperinsulinism/hyperammonemia syndrome. Rev Endocr Metab Disord.

[31] Jeffery CJ (2005). Mass spectrometry and the search for moonlighting proteins. Mass Spectrom Rev.

[32] Li C, Chen P, Palladino A (2010). Mechanism of hyperinsulinism in short-chain 3-hydroxyacyl-CoA dehydrogenase deficiency involves activation of glutamate dehydrogenase. J Biol Chem.

[33] Galcheva S, Demirbilek H, Al-Khawaga S, Hussain K (2019). The genetic and molecular mechanisms of congenital hyperinsulinism. Front Endocrinol.

[34] Stanley CA (2011). Two genetic forms of hyperinsulinemic hypoglycemia caused by dysregulation of glutamate dehydrogenase. Neurochem Int.

[35] Heslegrave AJ, Kapoor RR, Eaton S (2012). Leucine-sensitive hyperinsulinaemic hypoglycaemia in patients with loss of function mutations in 3-hydroxyacyl-CoA dehydrogenase. Orphanet J Rare Dis.

[36] Ben-Shushan E, Marshak S, Shoshkes M, Cerasi E, Melloul D (2001). A pancreatic beta -cell-specific enhancer in the human PDX-1 gene is regulated by hepatocyte nuclear factor 3beta (HNF-3beta ), HNF-1alpha, and SPs transcription factors. J Biol Chem.

[37] Sund NJ, Vatamaniuk MZ, Casey M (2001). Tissue-specific deletion of Foxa2 in pancreatic beta cells results in hyperinsulinemic hypoglycemia. Genes Dev.

[38] Vajravelu ME, Chai J, Krock B (2018). Congenital Hyperinsulinism and Hypopituitarism Attributable to a Mutation in FOXA2. J Clin Endocrinol Metab.

[39] De León-Crutchlow DD, Stanley CA. Congenital hyperinsulinism: a practical guide to diagnosis and management. Springer; 2019.

[40] Babiker O, Flanagan SE, Ellard S, Al Girim H, Hussain K, Senniappan S (2015). Protein-induced hyperinsulinaemic hypoglycaemia due to a homozygous HADH mutation in three siblings of a Saudi family. J Pediatr Endocrinol Metab JPEM.

[41] Kapoor RR, James C, Flanagan SE, Ellard S, Eaton S, Hussain K (2009). 3-Hydroxyacyl-coenzyme A dehydrogenase deficiency and hyperinsulinemic hypoglycemia: characterization of a novel mutation and severe dietary protein sensitivity. J Clin Endocrinol Metab.

[42] Stanley CA (2016). Perspective on the genetics and diagnosis of congenital hyperinsulinism disorders. J Clin Endocrinol Metab.

[43] Demirbilek H, Hussain K (2017). Congenital hyperinsulinism: diagnosis and treatment update. J Clin Res Pediatr Endocrinol.

[44] Kane C, Lindley KJ, Johnson PR, et al. Therapy for persistent hyperinsulinemic hypoglycemia of infancy. Understanding the responsiveness of beta cells to diazoxide and somatostatin. J Clin Investig 1997;100(7): 1888–93. 10.1172/JCI119718. 10.1172/JCI119718 PMC508376 9312191

[45] Çamtosun E, Flanagan SE, Ellard S, et al. A Deep Intronic HADH Splicing mutation (c.636+471G>T) in a congenital hyperinsulinemic hypoglycemia case: long term clinical course. J Clin Res Pediatr Endocrinol 2015;7(2): 144–7. 10.4274/jcrpe.1963. 10.4274/jcrpe.1963 PMC4563187 26316438

[46] Herrera A, Vajravelu ME, Givler S (2018). Prevalence of adverse events in children with congenital hyperinsulinism treated with diazoxide. J Clin Endocrinol Metab.

[47] Yorifuji T, Horikawa R, Hasegawa T (2017). Clinical practice guidelines for congenital hyperinsulinism. Clin Pediatr Endocrinol Case Rep Clin Investig.

[48] Thorton PS, Alter CAJTJop. Short-and long-term use of octreotide in the treatment of congenital hyperinsulinism. J Pediatr 1993;123(4): 637–43. 10.1016/s0022-3476(05)80969-2. 10.1016/s0022-3476(05)80969-2 8410522

[49] Hosokawa Y, Kawakita R, Yokoya S (2017). Efficacy and safety of octreotide for the treatment of congenital hyperinsulinism: a prospective, open-label clinical trial and an observational study in Japan using a nationwide registry. Endocr J.

[50] Eichmann D, Hufnagel M, Quick P, Santer R (1999). Treatment of hyperinsulinaemic hypoglycaemia with nifedipine. Eur J Pediatr.

